# A Self‐Adaptive Reconfigurable Metasurface for Electromagnetic Wave Sensing and Dynamic Reflection Control

**DOI:** 10.1002/advs.202505155

**Published:** 2025-06-11

**Authors:** Bo‐Wen Ren, Chu Qi, Peixing Li, Xiaoluo He, Alex M. H. Wong

**Affiliations:** ^1^ Department of Electrical Engineering City University of Hong Kong 83 Tat Chee Avenue Kowloon Hong Kong SAR China; ^2^ State Key Laboratory of Terahertz and Millimeter Waves City University of Hong Kong 83 Tat Chee Avenue Kowloon Hong Kong SAR China

**Keywords:** reconfigurable metasurface, self‐adaptive, sensing, dynamic control, applied electromagnetics

## Abstract

In the past decade, reconfigurable metasurfaces have attracted significant attention. While existing reconfigurable metasurfaces require human intervention to switch functionalities, feature non‐electromagnetic detection modalities or involve time‐consuming and resource‐intensive deep learning processes, in this work a simple real‐time self‐adaptive metasurface is reported that senses the direction of arrival of an incident wave, and adjusts its reflection accordingly. This proposed metasurface integrates sensing and reconfigurable reflection meta‐atoms using a simple phase comparator and a lookup table. This eliminates auxiliary detection modalities and external controller units, achieving dramatic savings in response time, energy consumption and fabrication cost. As examples, we design and investigated metasurfaces which self‐adaptively redirect or focus an incoming wave from any direction to a desired direction or location. It is experimentally demonstrated that the self‐adaptive metasurface can reflect the incoming wave from any direction within ±50° to the normal reflection direction with great efficacy. Further, the metasurface self‐adapts quickly, properly redirecting waves whose incidence angle changes by up to 12 deg/s with power consumption as low as 415 mW. Such metasurfaces provide an autonomous, computationally simple, cost‐effective, energy efficient and real‐time solution for a self‐adaptive reflection control, and open exciting possibilities for wireless communication, radar sensing and related applications.

## Graphical abstract

Reconfigurable metasurfaces typically require human intervention or complex, resource‐heavy processes. This innovative self‐adaptive metasurface senses an incident wave's direction, adjusting reflection in real‐time using a simple electronics module. It eliminates external controllers, reducing response time, energy consumption, and costs. Applications include efficiently redirecting or focusing waves, enhancing wireless communication and radar sensing capabilities.

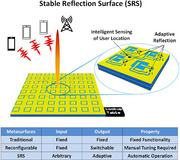

## 1 Introduction

The metasurface is a 2D metamaterial which exhibits user‐designed unconventional properties to manipulate electromagnetic (EM) waves. Compared to the traditional metamaterials, metasurfaces have attracted increasing attention from physicists and engineers due to their subwavelength thickness and lower fabrication complexity.^[^ ^1^, ^2^, ^3^, ^4^ ^]^ In the past decades, metasurfaces have found numerous applications, including beam steering,^[^ ^5^, ^6^ ^]^ negative refraction,^[^ ^7^, ^8^ ^]^ wave focusing,^[^ ^9^, ^10^ ^]^ and holography.^[^ ^11^, ^12^ ^]^ However, the traditional metasurfaces are limited by fixed functionality and lacking of dynamic control, which limits their applications.

To enhance the flexibility of traditional metasurfaces, many reconfigurable metasurfaces have been proposed for which the electromagnetic property of unit cells can be controlled optically, chemically, thermally, mechanically and/or electrically.^[^ ^13^, ^14^, ^15^, ^16^, ^17^ ^]^ Among these methods, electrical tuning has gained popularity for microwave and mm‐wave systems due, among other reasons, to their flexible control and easy integration with existing PCB‐based metasurfaces. Electrically reconfigurable metasurfaces are typically controlled by field programmable gate arrays (FPGAs) which send the required voltage biases to digitally switch between the “ON” and “OFF” states of PIN diodes, adjust the capacitances of varactors or change the material characteristics of phase change materials. Many reconfigurable metasurfaces have been reported with a wide range of functionalities including reconfigurable holography^[^ ^18^ ^]^ reflection‐transmission switching,^[^ ^19^, ^20^ ^]^ reconfigurable beam steering,^[^ ^21^, ^22^ ^]^ reconfigurable polarization conversion and vortex beam generation,^[^ ^23^, ^24^ ^]^ and reconfigurable focusing.^[^ ^25^ ^]^

**Figure 1:**
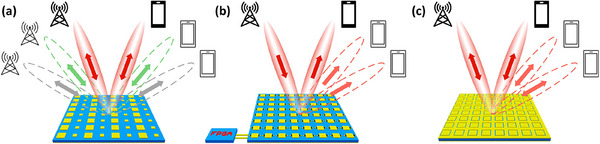
Applications of metasurfaces in wireless systems. a) Traditional fixed metasurfaces can achieve stable interactive communication between base stations and mobile stations with fixed locations. b) Reconfigurable metasurfaces can be controlled to adaptively reflect the signal from fixed base stations to multiple different locations. c) The proposed stable reflection surface (SRS) reflects signals from multiple mobile locations to a fixed location (e.g., a base‐station) by detecting the incoming directions and adapting its reflective properties.

**Figure** **1**a,b depict applications of the traditional fixed and reconfigurable metasurfaces in a wireless communication system. The anomalous reflection metasurface is used to illustrate the concept. The fixed metasurface (Figure 1a) can help redirect a signal from the transmitter into a desired direction, optimizing transmission efficiency and avoiding obstruction from barriers. However, due to its fixed functionality, the reflection direction for a fixed incident direction is also fixed, and barring specially engineered exceptions,^[^ ^7^ ^]^ incident signals from different directions will in general be anomalously reflected to different directions. On the other hand, the reconfigurable metasurface (Figure 1b) can reflect the signal from the same incident direction to multiple reflection directions by switching the metasurface state. As a result, multiple users located at different places can access the signal from a single transmitter in a time‐interleaved manner. Alternatively, signals sent from multiple users can be redirected to the same base‐station. However, to perform this switching one needs to know a priori the locations of the transmitter and receivers (i.e., the base‐station and end‐users), and pre‐programming which allows switching between the different channels.

Serving this need, self‐adaptive reconfigurable metasurfaces have been proposed where ambient conditions (such as gravity, velocities of objects, camera‐detected locations and temperature) are detected by auxiliary devices and used to adjust the metasurface's reflection response accordingly.^[^ ^26^, ^27^, ^28^, ^29^ ^]^ Recent studies on wideband programmable,^[^ ^30^, ^31^ ^]^ switchable cloaking,^[^ ^32^ ^]^ or intelligent sensing metasurfaces^[^ ^33^, ^34^, ^35^ ^]^ rely on auxiliary detection modalities (such as cameras, antennas and other sensors) and/or central computation units (FPGAs or computers), which increase their hardware and software complexities, energy costs and response times. Similarly, deep learning‐based self‐adaptive metasurfaces realize functionalities such as autonomous cloaking,^[^ ^36^ ^]^ focusing,^[^ ^37^ ^]^ power allocation^[^ ^38^ ^]^ in dynamically changing ambient environments. However, these metasurfaces need to collect a massive amount of environmental data to train the deep learning model onsite with human supervision, either before^[^ ^36^ ^]^ or during^[^ ^37^, ^38^ ^]^ their usage. Additionally, this involves significant computational resources and severely limits the metasurfaces’ response time, hence greatly reducing their adaptability to new environments. Therefore, a novel self‐adaptive metasurface, as shown in Figure 1c, that responds to the electromagnetics environment in real‐time, without a complex detection and response procedure, would be very attractive for wireless communication, radar and related applications. While Figure 1 depicts wireless communication with an anomalous reflection metasurface, self‐adaptive metasurfaces can find use also in imaging, sensing and energy harvesting and many other scenarios requiring dynamic tailoring of electromagnetic waves. These applications will benefit tremendously from a simple self‐responsive metasurface not involving auxiliary detection modalities and intensive computation (Figure 1c). A comparison of the three types of metasurfaces is listed in **Table** **1**.

**Table 1:** A comparison of the traditional metasurfaces, reconfigurable metasurfaces, and the proposed stable reflection surface (SRS).

Metasurfaces	Input	Output	Property	Control Complexity	Response Time	Energy Efficiency	External Device Requirement
Traditional	Fixed	Fixed	Fixed Functionality	Low	Fast	High	None
Reconfigurable (FPGA)	Fixed	Switchable	Manual Tuning Required	High	Moderate	Moderate	FPGA/ Computer
Reconfigurable (deep learning)	Fixed/ Arbitrary	Adaptive	Data‐Driven Adaptation	High	Slow	Low	FPGA/Computer/ Training Hardware
Proposed SRS	Arbitrary	Fixed and Stable	Autonomous Adaptation	Low	Fast	High	Lookup Table

In this paper, we propose a simple self‐adaptive reconfigurable metasurface that can detect the direction of an incoming wave without an auxiliary detection modality, then redirect the wave into the desired reflection direction without external controllers, such as a computer or an FPGA. The metasurface achieves hardware simplicity using minimal onboard components—a phase comparator and a lookup table (LUT)—to detect the direction‐of‐arrival (DoA) by sensing a small amount of the incident wave, unlike the complex hardware of FPGA‐based systems. The phase comparator‐LUT mechanism offers algorithm simplicity, relying on straightforward radiation direction‐phase distribution relations to adjust phase shifters via voltage biases, avoiding the intricate FPGA control and the complex data collection and training of deep learning approaches. This integrated design embeds sensing and self‐adaptive control within the metasurface, ensuring a system‐level autonomy, computational simplicity, and real‐time responsiveness for reflection control without external processing units. To illustrate the concept, we demonstrate a stable reflection metasurface (SRS), which reflects the incident wave to a fixed direction or focal point. The SRS achieves a DoA detection accuracy with a mean absolute error (MAE) of 1.18° and a maximum error margin of ±3°, and responds accurately to incident waves within the angular range from ‐50° to 50°, and remains stable under a dynamically varying incident angle with a fast angular velocity of up to 12 deg/s. Our SRS achieves reflection angle stabilization by autonomously adjusting phase shifts based on the incident angle, eliminating the need for real‐time reflection angle detection. The achievement of self‐adaptive reconfigurability without using an extra modality and computer control makes this metasurface simple, non‐power intensive, practical and attractive for applications in wireless communication and beyond.

## 2 Results

### 2.1 Design Overview of the Stable Reflection Surface

**Figure 2:**
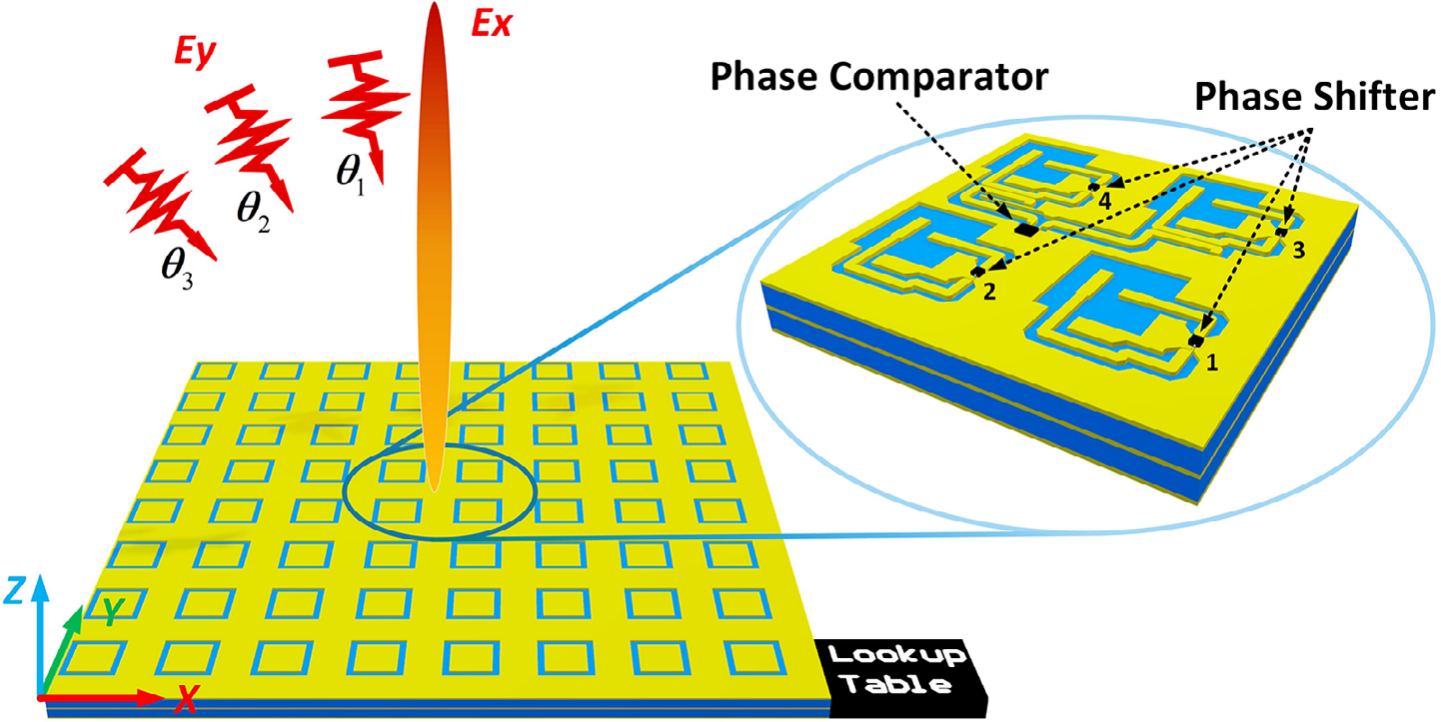
Schematic of the instinctive responsive stable reflection surface (SRS) concept. The SRS receives a *y*‐Pol incoming plane wave and reflects an *x*‐Pol wave with a required phase shift according to a lookup table, which maps the incident angle θ_*i*_ to a series of output voltage biases based on the predefined functionalities. θ_*i*_ is detected by a phase comparator chip. As an example, the figure demonstrates the functionality of reflecting incoming waves from arbitrary directions to the normal direction.

The schematic of the proposed SRS concept is depicted in **Figure** **2**. The SRS detects the incident angle of the incoming wave and rearranges the reflection phase to stabilize the reflection angle. The metasurface is equipped with a phase comparator and multiple phase shifter. The phase comparator detects the phase difference of the incoming wave onto two adjacent unit cells, from which the incident angle can be obtained. The required compensatory phase shift is applied on each unit cell, such that the desired reflection phase profile, and hence the reflection direction, is correspondingly adjusted.

The proposed SRS can be applied to steer the incident plane wave and stabilize the reflection direction. The wave propagation direction is related to the phase gradient on the metasurface. The SRS shown in Figure 2 reflects the incoming wave from arbitrary direction to the normal direction of the metasurface, i.e., θ_*r*_ =  0°. Specifically, phase shifters are tuned to compensate for the phase difference, such that the phases of reflected waves on all unit cells are identical. The required reflection phase for the *m*th unit cell can be expressed as:

(1) $$\varphi_{m}=\varphi_{0}-m\Delta \varphi_{xi}$$

in which the phase difference on two adjacent unit cells in *x*‐direction is expressed as:

(2) $$\Delta \varphi_{xi}=\frac{2\pi }{\lambda }D\sin\theta_{i}$$

The detailed derivations are shown in Sections S2, S3, Supporting Information. A metasurface implementing this phase shift will reflect the wave from arbitrary θ_*i*_ to θ_*r*_ =  0°. Theoretically, the proposed SRS can stabilize the reflection to any angle.

In addition, the SRS can also be applied to focus the incident plane wave to a stabilized focal point regardless of the incidence direction. The required reflection phase can be expressed as (see Sections S2,S3, Supporting Information):

(3) $$\varphi_{m}=\varphi_{0}-m\Delta \varphi_{xi}+\left(\sqrt{F^{2}+{\left(mD\right)}^{2}}-F\right)$$

in which *F* and *D* are the focal distance and unit cell size, respectively.

### 2.2 Unit Cell Design and Simulation

As is shown in Figure 2, the proposed SRS is composed of three metallic layers (yellow) and two intermediate substrate layers (blue). We assume the SRS is placed in *xy*‐plane of *z*  =  0, and select *x*‐direction as phase variation direction with *xz*‐plane to be the incident plane. Based on an example scenario of Bluetooth or Wi‐Fi, the operation frequency of 2.4 GHz is selected. The thickness and dielectric constant of both two substrate layers are 2 mm and 6.15, respectively. The top view of the SRS shows a 2‐dimensional array of patches surrounded by metallic region. The middle metallic layer is the ground plane. The main functionality is realized on the bottom metallic layer. The bottom view of the four representative unit cells in the center of the SRS is zoomed in and shown in the top right corner of the Figure 2, which are marked with numbers 1 to 4. Cells 1 and 2 are Type I (reflection) cells while Cells 3 and 4 are Type II (detection) cells. Both types of unit cells are equipped with a phase shifter chip for tuning the reflection phase. The Type II cell is slightly adjusted from Type I is connected to a phase comparator chip. Only Cells 3 and 4 on the metasurface are of Type II. All other unit cells (including those without numbers) are Type I.

**Figure 3:**
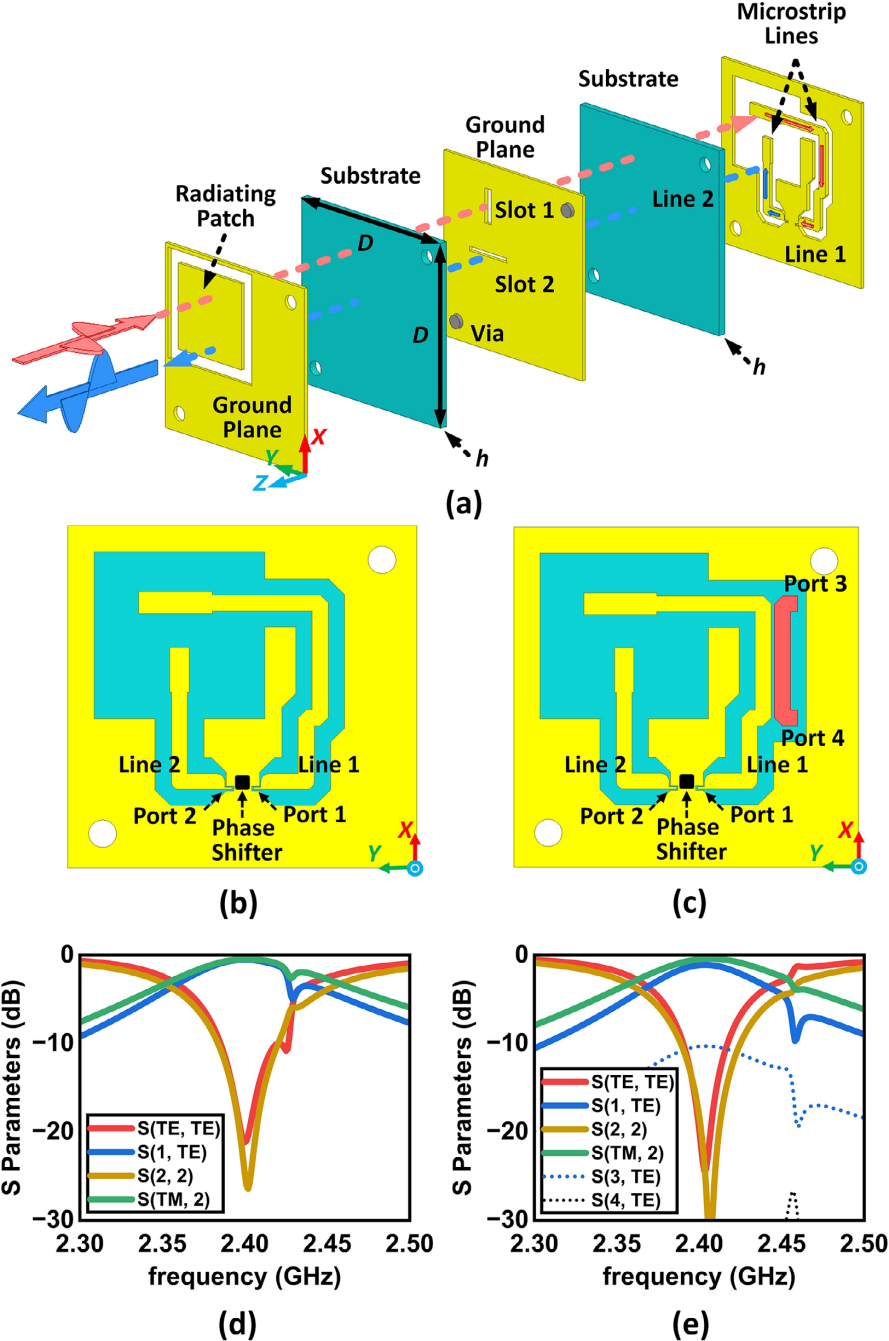
Topological view and simulation results of the unit cells. a) Stacked layer diagram of the proposed unit cells. The unit cells are composed of three metallic layers and two substrate layers. Both substrates have a thickness of 2 mm and a dielectric constant of 6.15, *D* = 50 mm. b, c) The bottom metallic layer for (b) Type I and (c) Type II unit cells. d, e) S‐parameters of the (d) Type I and (e) Type II unit cells within the bandwidth from 2.3 to 2.5 GHz.

The stacked view of the Type I unit cell is shown in **Figure** **3**a. **Table** **2** lists the geometric parameters. The two cells are the same on both the top and middle metallic layers but slightly different on bottom layer. As is shown in Figure 3a, the top metallic layer is composed of a square shaped radiating patch antenna surrounded by a grounded metal region. The bottom layer of the Type I cell, as shown in Figure 3b, contains two microstrip lines (labeled by Lines 1 and 2) terminated by Ports 1 and 2, which are preserved for the input and output ports of the phase shifter, respectively. The bottom structure of the Type II cell is similar to that of Type I cell, as shown in Figure 3c. An extra microstrip line (denoted in red) terminated by Ports 3 and 4 is placed in parallel to Line 1. The additional microstrip line couples with Line 1 and extracts 10% of signal power from Line 1 to Port 3. In the unit cell simulation, we terminate Port 3 with a lumped port. In the SRS, we route Ports 3 of two adjacent Type II cells to the phase comparator chip for phase comparison, which will allow us to determine the phase separation Δφ_*i*_ and in turn the DoA. The characteristic impedances of the microstrip lines are optimized to 50 Ω. The microstrip lines on the bottom surface are also surrounded by metallic ground regions. The middle metallic layer shown in Figure 3a is a ground plane for reflecting the waves. The two rectangular slots on the middle ground plane couple the wave between the patch on the top layer and the microstrip lines on the bottom layer. The metallic ground regions on the top and bottom layers are connected to the middle layer through vias to shield the active elements on the bottom layer from the incoming waves. The geometric details of all metallic layers are shown in Section S1 of Supporting Information.

**Table 2:** Comparison between FPGA and MCU.

Metrics	FPGA	MCU
Language	hardware description language	programming language
Compilation & Flashing Time	more than 1 min	less than 10 sec
Processing Mode	parallel	serial
Input/Output Mode	parallel	serial
Power Consumption	∼5 W	165 mW

The reflection phase is tuned by the phase shifter on the bottom surface. As is shown in Figure 3a, the patch structure in the top metallic layer receives a *y*‐Pol wave and reflects an *x*‐Pol wave. When the *y*‐Pol incident waves come in front of the radiating patch (+ *z* space), the fields couple to Line 1 on the bottom metal layer through Slot 1 and propagate to Port 1. The fields then travel through the phase shifter and into Line 2. Eventually, the fields couple again to the patch through Slot 2 and are reradiated. Since the two slots are perpendicular to each other, the reradiated fields are *x*‐polarized. For Type II unit cells (i.e., Cell 3 and 4), when the field is propagating along Line 1, 10% of the total power received is coupled to Port 3, while Port 4 is an isolated port. Since only a small part of the total power is extracted, the wave propagation and radiation for Cell 3 and 4 will not be affected seriously. Serving as a conceptual demonstration of the SRS, the co‐polarized incidence (*y*‐Pol) and cross‐polarized reflection (*x*‐Pol) design simplifies measurements by separating incident and reflected waves, as detailed in Section 2.4. While this introduces minor losses, a co‐polarized design could be achieved by aligning the microstrip lines to optimize efficiency in future iterations.

We simulate the unit cell using the commercial EM full‐wave simulation software Ansys High Frequency Structure Simulator (HFSS). Two Floquet ports are assigned to excite the unit cell, in which TE and TM denote the *y*‐Pol and *x*‐Pol incident field respectively. Figure 3d,e plot the S‐parameters of the Type I and Type II unit cells within the bandwidth from 2.3 to 2.5 GHz. For the Type I cell, S(1, TE) and S(TM, 2) denote the transmission coefficients from the incoming *x*‐Pol wave to Port 1 and from Port 2 to the radiated *y*‐Pol wave, respectively. S(TE, TE) and S(2, 2) denote the reflection coefficients of the incoming *x*‐Pol wave and Port 2, respectively. As is shown, the two transmission coefficients are around ‐0.5 dB, while the two reflection coefficients are lower than ‐20 dB. Thus, Ports 1 and 2 are well matched with low reflection, and that the cell, apart from the phase shifter, accrues a loss of less than 1 dB. For Type II cell, the additional S‐parameters S(3, TE) and S(4, TE) are transmission coefficients which denote coupling from the incoming *x*‐Pol wave to Port 3 and Port 4, respectively. As shown in Figure 3e and S(3, TE) is close to ‐10 dB as required in our design. The very low level of S(4, TE) implies that almost no power is lost to Port 4. This shows that both metasurface unit cells meet the design requirements. The simulation results of the unit cells at 2.4 GHz can be seen in Section S1 of Supporting Information.

### 2.3 SRS Simulation for Beam Steering and Focusing

**Figure 4:**
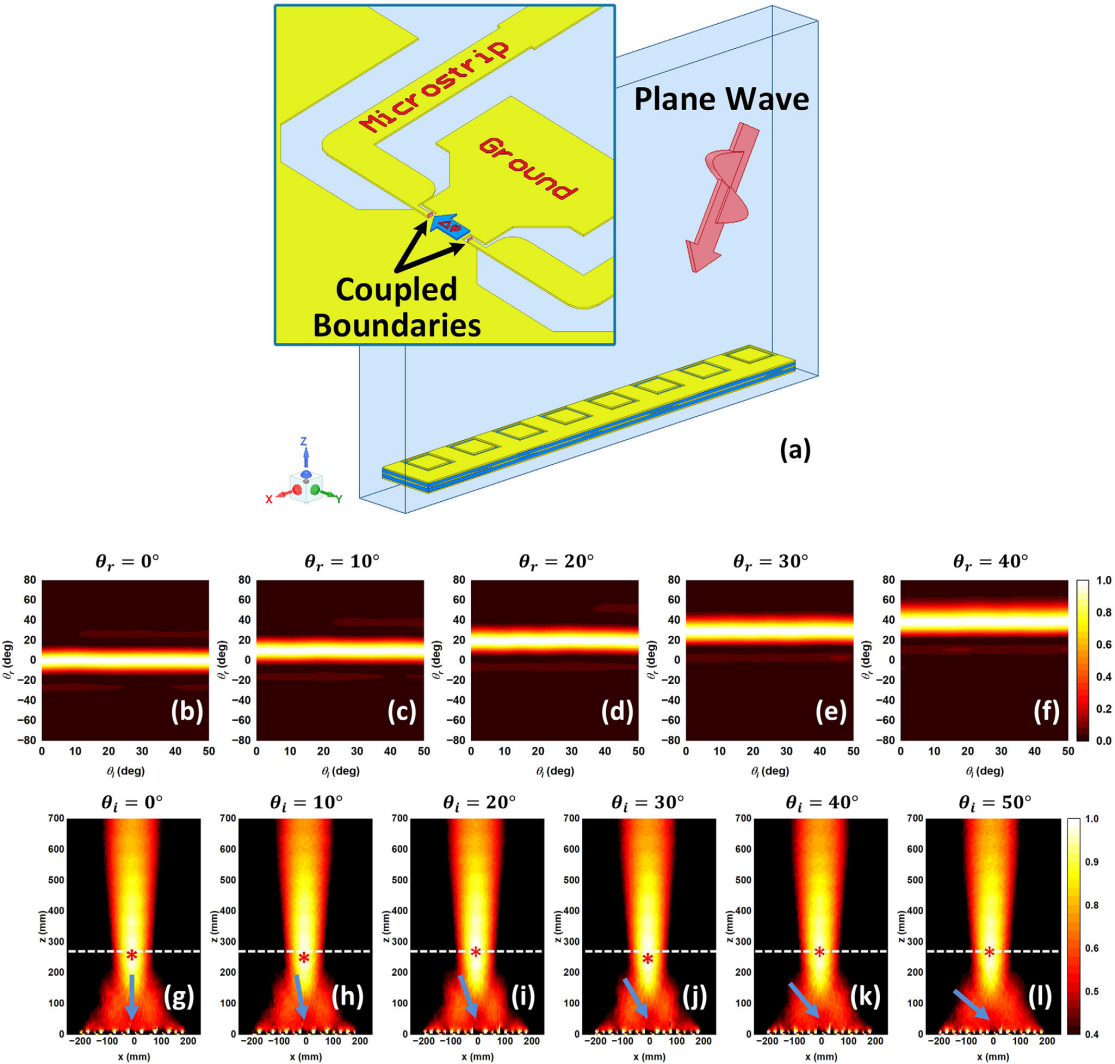
Simulation setup and field results of an 8 by 1 array. a) The SRS is placed in an air box and excited by an incident plane wave. Inset shows the close‐up view of part of the bottom surface, in which the two microstrip lines are terminated by two coupled boundaries with a phase shift Δφ to mimic the functionalities of the phase shifter chip. b‐l) Simulation results of the normalized reflection power of the SRS under multiple incidence angles for b‐f) beam steering and g–l) focusing functionalities. Blue arrows in f–k) show the incident wave directions for the focusing cases.

An 8 by 1 metasurface is simulated for beam steering and focusing. The simulation setup is shown in **Figure** **4**a. As is shown, the SRS is placed in a simulation box and excited by a plane wave in the *xz*‐plane with the incident angle θ_*i*_ varying from 0 to 50°. We use periodic boundaries (with no phase variation) on the ± *y*‐directions to simulate an infinite number of unit cells in the *y*‐direction. We terminate the ± *x* and ± *z* boundaries with radiation conditions. The inset in Figure 4a is a close‐up view of the bottom layer. To simulate the phase shifter, we terminate Ports 1 and 2 (which connect to the terminals of the phase shifter) by two coupled boundaries with the phase shift Δφ.

The simulation results of the SRS are shown in Figure 4b–l. The beam steering functionality for different reflection angles is shown in Figure 4b–f, which plots the reflected wave's scattering pattern (θ_*r*_, vertical axis) under different incidence directions (θ_*i*_, horizontal axis). Under each incident angle, the reflection pattern is normalized by the maximum power density. As shown, the main reflection beam appears in the designed direction, no matter where the incident wave comes from. The focusing functionality of the SRS with the designed focal length *F*  =  300 mm is shown in Figure 4g–l. The normalized reflected field intensity under different incident angles is plotted. The focal plane is marked by the white dashed line, while the location with the strongest electric field is highlighted by the red asterisk. It is shown that the strongest electric field appears on or very close to the focal plane, which validates the fact that the incident plane waves from any direction are reflected and focused at the designed position.

### 2.4 Experimental Verification

**Figure 5:**
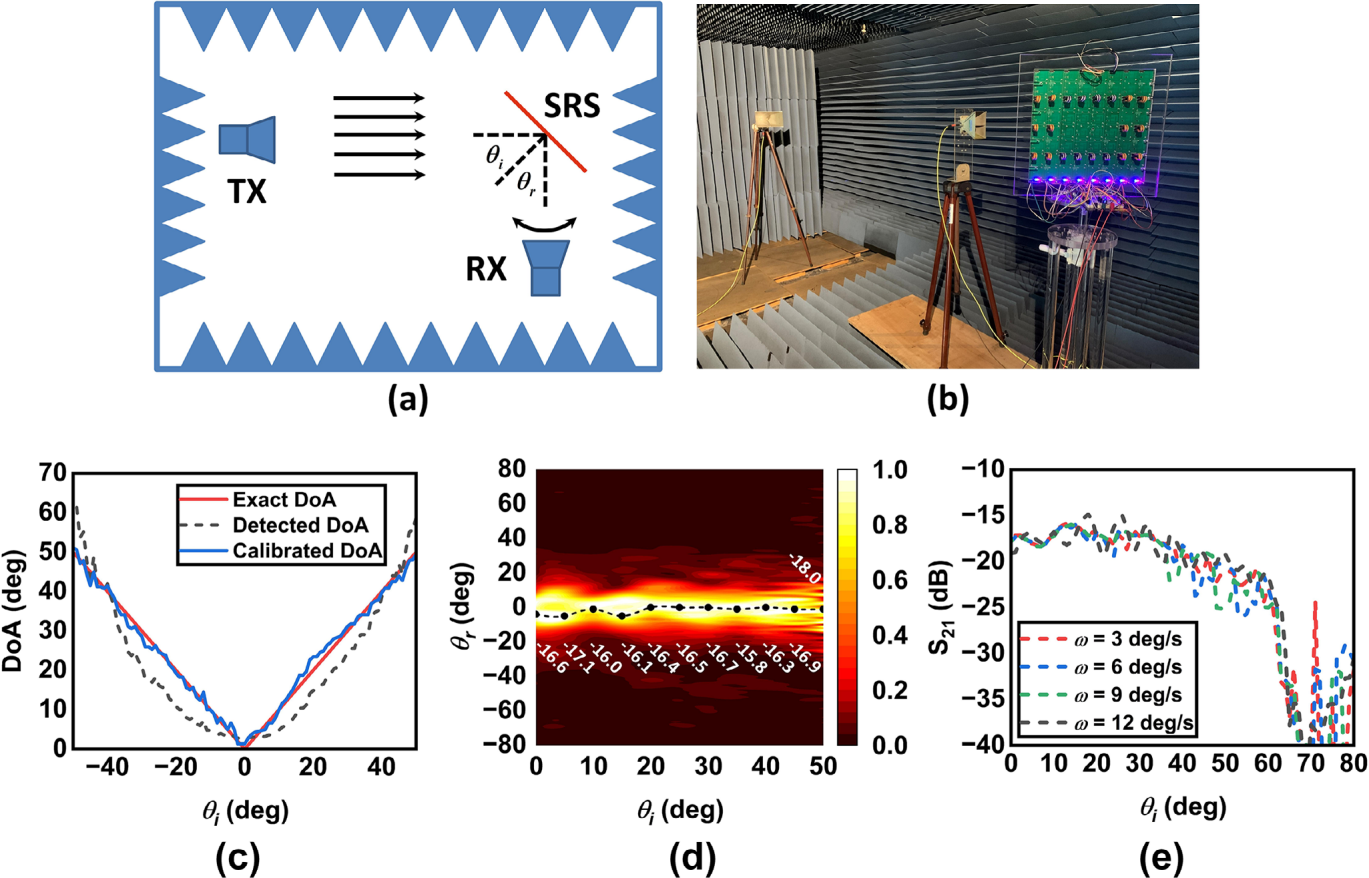
Experimental results of the fabricated normal reflection SRS. a) A schematic diagram and b) a photograph of the measurement setup. c) Comparison of exact DoA, detected DoA, and calibrated DoA for incident angles from ‐50° to +50°. The mean absolute error (MAE) between the calibrated and exact DoA is 1.18°, with a maximum error margin of ±3°. d) Normalized radiation pattern of the reflection field under different incident angles. The main beam remains at the normal direction (θ_*r*_ =  0°) when θ_*i*_ varies from 0° to 50°. e) Received power in the normal direction when sweeping the incidence angle (θ_*i*_) with multiple angular velocities. The received power maintains stable for incident angles lower than 50° when the angular velocity is below 12 deg/s.

We fabricate and test an 8 by 8 normal reflection SRS operating at 2.4 GHz to demonstrate its ability to perform self‐adaptive beam steering. The fabricated SRS uses a phase comparator (Analog Devices AD8302) to detect the phase difference between two Type II unit cells and adjusts phase shifters (M/A‐COM MAPS‐010164) via a LUT to stabilize the reflection direction. Detailed fabrication specifications, including the PCB substrate, phase shifter tunability, and control mechanisms, are provided in Section S4 of Supporting Information. The experimental setup, shown in **Figure** **5**a–b, involves a transmitting (Tx) and a receiving (Rx) horn in a microwave anechoic chamber. The Tx horn antenna is fixed in front of the SRS and generates a vertically polarized (V‐Pol) incident plane wave. The Rx horn antenna is fixed on a turn table and rotates around the SRS to measure the radiation pattern of the horizontally polarized (H‐Pol) reflected field. The entire setup resides in a microwave anechoic chamber to eliminate back scattering and electromagnetic interference. In this work, three measurements are implemented as described in the following subsections.

#### 2.4.1 Angle Detection Accuracy

We evaluated the DoA detection accuracy of the proposed SRS through detailed experimental measurements across an incident angle range of ‐50° to +50°. The phase comparator, utilizing the phase difference between adjacent Type II unit cells, forms the basis of our angle estimation. Experimental results, shown in Figure 5c, compare the exact DoA, detected DoA, and calibrated DoA. The detected DoA is calculated theoretically using Equation S7 (Supporting Information), while the calibrated DoA is obtained after curve‐fitting‐based calibration. The calibrated DoA, used in subsequent experiments, matches the exact DoA closely, with a mean absolute error (MAE) of 1.18° and a maximum error margin of ±3° across the tested range, reflecting the practical performance of our hardware‐based approach.

#### 2.4.2 Reflection Pattern of the Fabricated SRS under Multiple Incident Angles

To validate the normal reflection functionality of the fabricated metasurface, we measure the scattering patterns of the metasurface under a range of incident angles. Specifically, in each measurement, we fix θ_*i*_ and rotate the Rx horn antenna around the SRS to measure the reflected fields from a wide range of angles from − 80° to 80°. By applying multiple θ_*i*_’s, the reflection performance of the metasurface under different DoA is evaluated, which validates the beam steering functionality of the developed metasurface. This step is to verify the stabilization of the reflection, instead of part of the DoA estimation, which relies solely on phase comparison. Therefore, the detection of reflection angle will not introduce additional steps or complexity to the DoA estimation.

Figure 5d shows the normalized scattered power of the fabricated SRS as a function of θ_*i*_. The scattered power distributions are normalized by the maximum received power for each measurement. In addition, the locations of the maximum power are highlighted by the black dashed line, with the related maximum power ratio *S*_21_ labeled in dB. As shown, the peak value of the reflected field appears at ± 5° around the normal direction under all incidence angles. The slight direction fluctuation may be caused by the slight inaccuracy of the incident angle θ_*i*_ in the measurement setup. In addition, the wider reflection main beam for θ_*i*_ ranging from 0° to 10° is mainly due to feed blockage. The normal reflection is achieved for incident angles up to 50°. These results show that the metasurface can self‐adaptively adjust the phase distribution under unknown or varying incident angle to preserve the normal reflection functionality.

#### 2.4.3 Incident Angle Sweep of the Fabricated SRS for Normal Receiving

To test the respond speed of the SRS, we measure the normal reflection under a time‐varying incidence. We fix the Rx horn in the normal direction of the SRS (θ_*r*_ =  0°), while rotating the Tx horn from 0 to 90° with a constant rotation speed ω  = dθ_*i*_/d*t* . The measurement is repeated with multiple rotation speeds to test the self‐adjustment ability and response speed. Since the phase comparator chip is measuring the incident angle all the time, the phase distribution for each unit cell would update accordingly. As a result, the scattering power will be kept in the normal direction as long as the metasurface's electronic response speed follows the steering speed of the incident wave.

Figure 5e plots the *S*_21_, which can be interpreted as the relative received power under the same incident power. When the angular velocity ω is at 9 deg/s or slower, the power ratio in the normal direction remains at a high level until the incident angle increases to 60°. The received power starts to decrease when the incident angle further increases due to the large angle oblique incidence. In fact, the received power shown in Figure 5g under different incident angles is close to the corresponding peak values in Figure 5f. When ω becomes 12 deg/s, the curve starts to show obvious fluctuation, which is caused by the shaking of the fast‐rotating turntable. However, the received power still remains a high level under incident angle within 50°. A slight roll‐off in power is attributed to the reduction in projected aperture. The results validate the dynamic control performance of the designed SRS under varying EM environment. The SRS's response time (see Section S5, Supporting Information), comprising 15 ns for phase comparison, 0.66 ns for signal propagation, and 1 µs for ADC/LUT processing, totals ≈1.02 µs. This enables real‐time adaptation at 12°/s and supports theoretical stability up to ≈10^6^ deg/s, with a practical limit of ≈2000 deg/s, suitable for applications like wireless communication and radar.

In addition, the reflection performance of proposed SRS in comparison with that of a piece of PEC and EM absorber is shown in Videos S7–S9, Supporting Information. In the three videos, the SRS, PEC and EM absorber are excited by a stationary Tx horn, while the metasurface and the Rx horn directly facing the metasurface are rotated at a speed of ω  =   − 3 deg/s. This effectively measures, in a modified frame of reference, broadside reflection when the Tx horn rotates with ω  =  3 deg/s, from 0° to 40°. The VNA screen in the left half scene displays the relative received power in real‐time. The right half scene shows the rotation of the three materials in measurement, which is synchronized to the VNA display. As is shown, the received power from the proposed SRS remains at around ‐20 dB, which is 10 dB higher than that from the PEC and EM absorber. Specifically, the PEC will reflect the incident wave to the specular direction, while the absorber will absorb it. Therefore, almost no power can be received in the normal direction of the material. In conclusion, the proposed SRS realizes the self‐adaptive reflection direction control to stabilize the high received power in the desired position.

## 3 Discussion and Conclusion

The technical performance of the self‐adaptive stable reflection surface (SRS) is evaluated. Our experiments have demonstrated that the SRS achieves robust normal reflection for incident angles from ‐50° to +50°, and can self‐adapt to changing incidence directions with angular velocities up to 12 deg/s, validating its real‐time autonomous performance. The SRS achieves rapid performance through a streamlined direction‐of‐arrival (DoA) detection and lookup table (LUT) response, eliminating external controller hardware. Its phase comparator‐LUT mechanism simplifies algorithms by using direct radiation direction‐phase relationships to adjust phase shifters via voltage biases, bypassing complex FPGA programming and data‐intensive deep learning processes. The SRS achieves DoA detection with a mean absolute error (MAE) of 1.18° and an error margin of ±3°, which is sufficient for stabilizing the reflection angle, meeting wireless communication and radar system needs. Unlike existing complex techniques,^[^ ^33^, ^34^ ^]^ our approach minimizes computational overhead, suiting resource‐constrained applications despite slightly lower precision. DoA detection accuracy can be further improved by increasing the number of unit cells for phase comparison (Li et al.,^[^ ^33^ ^]^ error < 1.5°). Rather than optimizing solely for DoA estimation, our design integrates DoA detection with the novel SRS functionality for adaptive reflection control, providing an autonomous, hardware‐efficient solution. The SRS achieves a low power consumption of 415 mW, with an average power per area of 2.6×10^−4^ W cm^−2^, outperforming existing reconfigurable metasurfaces, such as Ma et al. (1.4×10^−2^ W cm^−2^)^[^ ^39^ ^]^ and Wang et al. (5.1×10^−4^ W cm^−2^),^[^ ^40^ ^]^ ideal for autonomous operation. This efficiency results from its simple structure and control logic, eliminating power‐intensive components like FPGAs or external sensors. The microcontroller unit (MCU)‐based system (Table 2) outperforms FPGA‐based designs in response time and programming simplicity, ensuring autonomy, real‐time responding and energy efficiency without auxiliary hardware.

Future work will enhance the SRS's performance and extend its capabilities. An advanced experimental setup with faster and more stable turntable will enable the measurement of angular velocities beyond 12 deg/s. Broadband, large‐angle unit cells, such as magnetoelectric dipole designs such as magnetoelectric dipole element^[^ ^41^ ^]^ will enhance bandwidth and incident angle range. Orthogonal phase comparison and independent phase control will enable 2D DoA detection, beam steering and focusing, as validated by simulations (Figures S2 and S3, Supporting Information). Additionally, integrating adaptive lookup table optimization will further increase flexibility for diverse applications.

In summary, our metasurface achieves real‐time DoA detection and reflection control with minimal complexity, surpassing FPGA and deep learning methods. It enables rapid, reliable beam steering for next‐generation wireless communication, especially in mobile‐to‐fixed receiver scenarios. By stabilizing reflection without external intervention, it offers a practical solution for encrypted communication and energy‐efficient networks, outperforming traditional complex control systems.

## Conflict of Interest

The authors declare no conflict of interest.

## Supporting information



Supporting Information

Supplementary Video

Supplementary Video

Supplementary Video

## Data Availability

The data that support the findings of this study are available from the corresponding author upon reasonable request.
