# Supplementary material for: A Self‐Adaptive Reconfigurable Metasurface for Electromagnetic Wave Sensing and Dynamic Reflection Control
Source: Adv Sci (Weinh). 2025 Jun 11;12(32):e05155. doi: 10.1002/advs.202505155 (PMC12407367; doi:10.1002/advs.202505155)
Supplement: Supplementary file 1 — Supporting Information [file ADVS-12-e05155-s002.pdf]

ADVANCED SCIENCE
Open Access

Supporting Information

for *Adv. Sci.*, DOI 10.1002/advs.202505155

A Self-Adaptive Reconfigurable Metasurface for Electromagnetic Wave Sensing and Dynamic Reflection Control

*Bo-Wen Ren, Chu Qi, Peixing Li, Xiaoluo He and Alex M. H. Wong**

Supporting Information for

A Self-adaptive Reconfigurable Metasurface for Electromagnetic Wave Sensing and Dynamic Reflection Control

*Bo-Wen REN, Chu QI, Peixing LI, Xiaoluo HE, and Alex M. H. WONG**

B. W. REN, C. QI, P. LI, X. HE, A. M. H. WONG
Department of Electrical Engineering
City University of Hong Kong
Hong Kong SAR, China
E-mail: alex.mh.wong@cityu.edu.hk
A. M. H. WONG
State Key Laboratory of Terahertz and Millimeter Waves
City University of Hong Kong
Hong Kong SAR, China

The Supporting Information includes:

S1. Geometric Details of the Unit Cells and Fabricated Stable Reflection Surface (SRS)
S2. Two-Dimensional Direction of Arrival (DoA) Detection
S3. Two-Dimensional Reflection Beam Steering and Focusing
S4. Fabricated SRS and Control Mechanisms
S5. Responding Time Analysis
S6. Power Consumption Analysis
S7. Reflection of the proposed SRS under Varying Incident Angle
S8. Reflection of a Piece of Planar Aluminum Board under Varying Incident Angle
S9. Reflection of a Piece of Microwave Absorber under Varying Incident Angle

S1. Geometric Details of the Unit Cells and Fabricated Stable Reflection Surface (SRS)

The geometric details of the unit cells are shown in **Figure S1**a-d and Table S1. The simulation results of the unit cells at 2.4 GHz are reported in **Table S2**.

Table S1. Geometric parameters of the proposed unit cell.

Parameter	D	h	W_T	W_1	W_P	L_P	W_{s1}	W_{s2}	L_{s1}	L_{s2}
Value (mm)	50.0	2.0	2.3	30.0	22.6	23.3	2.2	1.3	10.6	6.6

Table S2. Scattering *S* Parameters of two types of unit cells at 2.4 GHz.

S Parameters	Type I Cell	Type II Cell
S(TE, TE)	-20.88 dB	-19.28 dB
S(2, 2)	-24.33 dB	-18.05 dB
S(TM, 2)	-0.43 dB	-0.47 dB
S(1, TE)	-0.51 dB	-1.14 dB
S(3, TE)	N/A	-10.30 dB
S(4, TE)	N/A	-37.33 dB

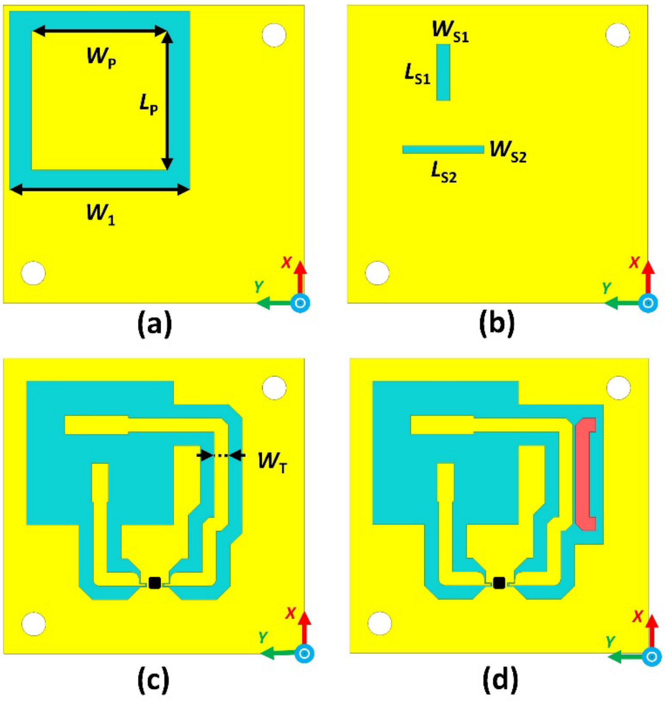

Figure S1. Topological view of the metallic layers of the unit cells. (a) The top metallic layer. (b) The middle metallic layer. (c-d) The bottom metallic layer for (c) Type I and (d) Type II unit cells.

S2. Two-Dimensional Direction of Arrival (DoA) Detection

This note provides a detailed derivation of the phase differences detected by a two-dimensional metasurface receiving an incoming plane wave, enabling direction of arrival (DoA) estimation. The metasurface is composed of unit cells with a uniform spacing of D in both x- and y-directions. An incoming plane wave is characterized by an azimuthal angle ϕ_i, elevation angle θ_i, and frequency f. The wave vector $\vec{k}$ can be expressed in Cartesian coordinate system as follows:

$$\vec{k} = k(\hat{x}\sin\theta_i \cos\phi_i + \hat{y}\sin\theta_i \sin\phi_i - \hat{z}\cos\theta_i) \tag{S1}$$

in which the wave number $k = \frac{2\pi}{\lambda}$. This wave vector describes the direction of propagation, with the negative z-component indicating incidence from the upper half-space toward the metasurface located in the xy-plane ($z = 0$). The phase difference between two adjacent unit cells is determined by the projection of the wave vector onto the displacement vectors between cells. The phase difference on two adjacent unit cells in x- and y-directions can be expressed in terms of:

$$\Delta\varphi_{xi} = \frac{2\pi}{\lambda}\vec{k}\cdot(D,0,0) = \frac{2\pi}{\lambda}D\sin\theta_i \cos\phi_i \tag{S2}$$

$$\Delta\varphi_{yi} = \frac{2\pi}{\lambda}\vec{k}\cdot(0,D,0) = \frac{2\pi}{\lambda}D\sin\theta_i \sin\phi_i \tag{S3}$$

The phase differences for the proposed SRS ($D = 50$ mm, $\lambda = 125$ mm) can be calculated. The results for some typical pairs of (ϕ_i, θ_i) are shown in Table S3. The azimuthal angles cover cases for x-plane ($\phi_i = 0°$), y-plane ($\phi_i = 90°$), and arbitrary ($\phi_i = 30°, 45°$) incidence. In addition, the elevation angles cover 0° to 50°, which is consistent with the acceptable incident angle range $\pm 50°$ of the proposed SRS. With the same elevation angle θ_i, the values of $\Delta\varphi_{xi}$ and $\Delta\varphi_{yi}$ are exchanged for x-plane ($\phi_i = 0°$) and y-plane ($\phi_i = 90°$) incidence. This is caused by the phase gradient symmetry of the incident wave along the plane of $\phi_i = 45°$, which is also consistent with the expression of $\Delta\varphi_{xi}$ and $\Delta\varphi_{yi}$ Equation S2 and S3. Additionally, it can be observed that $\Delta\varphi_{xi} = \Delta\varphi_{yi}$ when $\phi_i = 45°$. The calculated pairs of $(\Delta\varphi_{xi}, \Delta\varphi_{yi})$ are applied to the two-dimensional simulations of the proposed SRS, as shown in Section 3.3.

Table S3. Phase differences for some typical pairs of (ϕ_i, θ_i).

(ϕ_i, θ_i)	$(\Delta\varphi_{xi}, \Delta\varphi_{yi})$	(ϕ_i, θ_i)	$(\Delta\varphi_{xi}, \Delta\varphi_{yi})$
$(0°, 0°)$	$(0°, 0°)$	$(30°, 0°)$	$(0°, 0°)$
$(0°, 25°)$	$(60.86°, 0°)$	$(30°, 25°)$	$(52.70°, 30.43°)$
$(0°, 50°)$	$(110.31°, 0°)$	$(30°, 50°)$	$(95.53°, 55.16°)$
$(90°, 0°)$	$(0°, 0°)$	$(45°, 0°)$	$(0°, 0°)$
$(90°, 25°)$	$(0°, 60.86°)$	$(45°, 25°)$	$(43.03°, 43.03°)$
$(90°, 50°)$	$(0°, 110.31°)$	$(45°, 50°)$	$(78.00°, 78.00°)$

In terms of $\Delta\varphi_{xi}$ and $\Delta\varphi_{yi}$, the phase of the incident wave on the unit cell at the location $(mD, nD, 0)$ can be expressed as:

$$\varphi_{Imn} = m\Delta\varphi_{xi} + n\Delta\varphi_{yi} \tag{S4}$$

Based on the detected phase differences $\Delta\varphi_{xi}$ and $\Delta\varphi_{yi}$, the estimated azimuthal angle ϕ_i and elevation angle θ_i of the incident plane wave can be expressed as:

$$\theta_i = \sin^{-1}\left[\frac{\lambda}{2\pi D}\sqrt{\Delta\varphi_{xi}^2 + \Delta\varphi_{yi}^2}\right] \tag{S5}$$

$$\phi_i = \tan^{-1}\frac{\Delta\varphi_{yi}}{\Delta\varphi_{xi}} \tag{S6}$$

In case of one-dimensional DoA extimation in x-direction, as described in the main text (Figure 1), $\Delta\varphi_{yi} = 0$. Therefore,

$$\theta_i = \sin^{-1}\left[\frac{\lambda}{2\pi D}\Delta\varphi_{xi}\right] \tag{S7}$$

$$\phi_i = 0 \tag{S8}$$

These phase differences are measured by the phase comparator in Type II unit cell, as described in the main text (Figure 2).

S3. Two-Dimensional Reflection Beam Steering and Focusing

S3.1 Beam Steering

For a two-dimensional reflective metasurface with $M \times N$ unit cells, the main radiation direction of the reflection beam is dominated by the array factor (AF):

$$AF(\theta_r, \phi_r) = \sum_m \sum_n e^{j[k(mD\sin\theta_r\cos\phi_r + nD\sin\theta_r\sin\phi_r) + \varphi_{Smn}]} \tag{S9}$$

in which the phase φ_{Smn} of the unit cell at the location $(mD, nD, 0)$ can be expressed in terms of the reflected phase difference on two adjacent unit cells in x- and y-directions:

$$\varphi_{Smn} = \varphi_{00} + m\Delta\varphi_{xr} + n\Delta\varphi_{yr} \tag{S10}$$

in which φ_{00} denotes the reflected phase of the unit cell at the location $(0, 0, 0)$. In the main radiation beam direction (θ_r, ϕ_r), the phase of each term in Equation S5 should be zero, which means

$$k(mD \sin\theta_r \cos\phi_r + nD \sin\theta_r \sin\phi_r) + \varphi_{Smn} = 0 \tag{S11}$$

from which we can obtain

$$kD \sin\theta_r \cos\phi_r + \Delta\varphi_{xr} = 0 \tag{S12}$$

$$kD \sin\theta_r \sin\phi_r + \Delta\varphi_{yr} = 0 \tag{S13}$$

Therefore, to obtain maximum radiation intensity in direction (θ_r, ϕ_r), the required phase difference on two adjacent unit cells in x- and y-directions are

$$\Delta\varphi_{xr} = -kD \sin\theta_r \cos\phi_r \tag{S14}$$

$$\Delta\varphi_{yr} = -kD \sin\theta_r \sin\phi_r \tag{S15}$$

in which the wave number $k = \frac{2\pi}{\lambda}$. Therefore, to reflect the incident wave from direction (θ_i, ϕ_i) to direction (θ_r, ϕ_r), the required reflection phase of unit cell at the location $(mD, nD, 0)$ can be expressed as:

$$\varphi_{mn} = \varphi_{Smn} - \varphi_{Imn} = \varphi_{00} + m(\Delta\varphi_{xr} - \Delta\varphi_{xi}) + n\left(\Delta\varphi_{yr} - \Delta\varphi_{yi}\right) \tag{S16}$$

in which $\Delta\varphi_{xi}$ and $\Delta\varphi_{yi}$ are expressed in Equation S2 and S3, $\Delta\varphi_{xr}$ and $\Delta\varphi_{yr}$ are expressed in Equation S14 and S15. For beam steering in x-direction, $\phi_r = \phi_i = 0$. As a result, Equation S12 becomes:

$$\varphi_m = \varphi_0 + m(\Delta\varphi_{xr} - \Delta\varphi_{xi}) \tag{S17}$$

Specifically, for normal reflection, $\theta_r = 0$, which leads to:

$$\varphi_m = \varphi_0 - m\Delta\varphi_{xi} \tag{S18}$$

S3.2 Beam Focusing

For a metasurface in the xy-plane with unit cells spaced by D, beam focusing to a focal point $(0, 0, F)$, requires a hyperbolic phase profile. As established in metasurface optics[1], the reflection phase of unit cell at the location $(mD, nD, 0)$ is:

$$\varphi_{Fmn} = \varphi_{00} + \frac{2\pi}{\lambda}\left(\sqrt{F^2 + (m^2 + n^2)D^2} - F\right) \tag{S19}$$

Similarly, to focus the incident wave from direction (θ_i, ϕ_i) to the focal point $(0,0,F)$, the required reflection phase of unit cell at the location $(mD, nD, 0)$ can be expressed as:

$$\varphi_{mn} = \varphi_{Fmn} - \varphi_{Imn} = \varphi_{00} - m\Delta\varphi_{xi} - n\Delta\varphi_{yi} + \frac{2\pi}{\lambda}\left(\sqrt{F^2 + (m^2 + n^2)D^2} - F\right) \tag{S20}$$

For one-dimensional beam focusing in x-direction, $\phi_i = 0$:

$$\varphi_m = \varphi_0 - m\Delta\varphi_{xi} + \frac{2\pi}{\lambda}\left(\sqrt{F^2 + (mD)^2} - F\right) \tag{S21}$$

S3.3 Simulation Results of two-dimensional DoA detection and reflection

To demonstrate the feasibility of 2D wavefront manipulation, we conducted simulations using a 4×4 metasurface with independent phase control in both x- and y-directions, reducing computational demands compared to an 8×8 array. As shown in **Figure S2**, we evaluate 2D direction of arrival (DoA) detection and beam steering pattern for incident angles $\theta_i = 0°, 25°$, and $50°$ with reflection angles $\theta_r = 0°$ and $40°$ in both xz- and yz-planes ($\phi_i = 0°$ and $90°$, respectively). To further illustrate versatility, we tested cases where the incident and reflected waves are not coplanar, with azimuthal angles $\phi_i = 30°$ and $\phi_r = 60°$ for the same θ_i and θ_r values. The calculated phase differences $(\Delta\varphi_{xi}, \Delta\varphi_{yi})$ are shown in Table S3. The radiation patterns shown in Figure S2 confirm successful 2D beam steering, with angle errors of less than $2°$ for both azimuthal ϕ_r and elevation θ_r angles across all tested conditions.

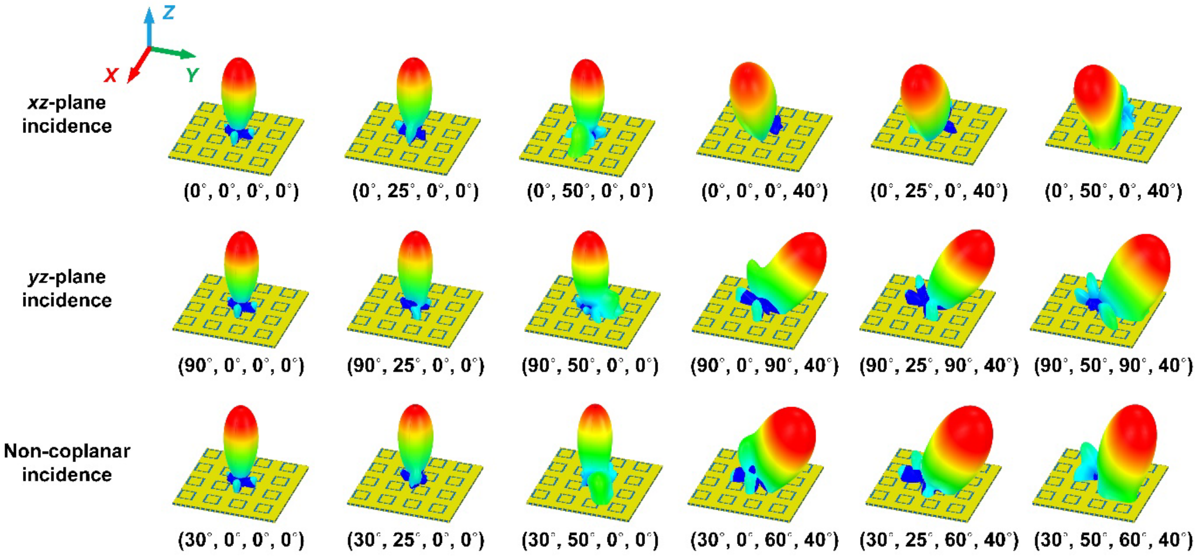

Figure S2. Beam steering patterns of a simulated 4×4 SRS under multiple incident and reflection conditions. The four values $(\phi_i, \theta_i, \phi_r, \theta_r)$ denote the azimuthal and elevation angles of the incident and reflection waves, respectively.

In **Figure S3**, we evaluate 2D beam focusing results for incident angles $\theta_i = 0°$, 25°, and 50° with $\phi_i = 0°$, 45° and 90°. The calculated phase differences $(\Delta\varphi_{xi}, \Delta\varphi_{yi})$ are shown in Table S3. Since 4×4 array is simulated for 2D demonstration, we design the focal length as $F = 150$ mm, which is half of that of the 8×1 array shown in the main text, to remain the numerical aperture. The normalized electric field (E field) focused around the focal length shown in Figure S3 confirms successful 2D beam focusing performances. The strong near field and relative weak intensity at the focal point are caused by the small metasurface size and focal length, which can be improved in practice by increasing the unit cell number and focal length F.

These results validate the theoretical viability of our SRS for 2D DoA detection and beam steering with minimal performance degradation compared to 1D operation. The computational overhead remains manageable, as phase adjustments are handled by a lookup table (LUT) based on phase differences, preserving the simplicity of our MCU-based system (Section 4). These simulations indicate that our SRS design is readily generalizable to 2D applications.

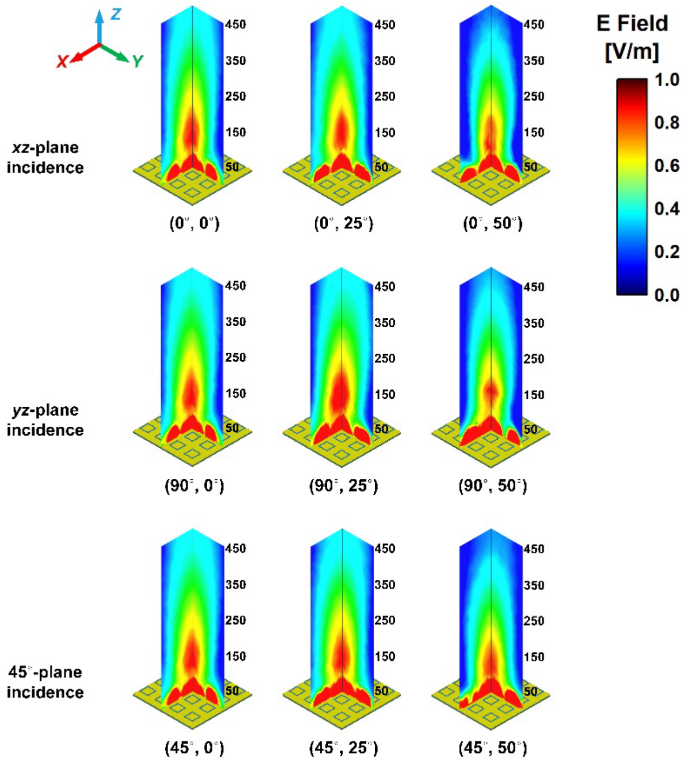

Figure S3. Normalized focusing electric field (E field) results of a simulated 4×4 SRS under multiple incident conditions. Designed focal length $F = 150$ mm. The two values (ϕ_i, θ_i) denote the azimuthal and elevation angles of the incident waves, respectively. The position values in z-axis are expressed in the unit of mm.

S4. Fabricated SRS and Control Mechanisms

The fabricated normal reflection SRS with 8 by 8 unit cells is shown in **Figure S4**. The SRS is comprised mostly of Type I unit cells. Two Type II unit cells, included near the center for direction sensing, are labelled in the figure. The SRS is composed of two stacked printed circuit boards (PCB) fixed by steel screws. We select the F4B material with relative dielectric constant ε_r of 6.15 and thickness of 2 mm as the PCB substrate. A wideband RF phase shifter (M/A-COM MAPS-010164, 2.3-3.8 GHz) is soldered to Ports 1 and 2 on the unit cell's bottom surface. This phase shifter has 6-bit digital phase tunability which allows one to precisely tune the phase between the input and output RF ports. We express the 6-digit input of the phase shifter in terms of $[B_{\text{in}}]_2$, in which the subscript 2 denotes the binary number. Each digit is "0" or "1", denoting the required voltage level of 0 V and 5 V, respectively. According to the datasheet[2], the phase $\angle S_{21}$ of the transmission coefficient from the RF input to output ports of the chip is related to B_{in} governed by

$$\angle S_{21}\ [\text{deg}] = 5.625[B_{\text{in}}]_{10}\ , \qquad \text{(S22)}$$

in which $[B_{\text{in}}]_{10}$ is the decimal expression of B_{in}. For example, if the 6 input ports of the chip are provided with "0 V, 5 V, 0 V, 5 V, 0 V, 5 V", the detected $[B_{\text{in}}]_2$ is "010101", or $[B_{\text{in}}]_{10} = 21$. According to Equation 4, the obtained $\angle S_{21} = 118.125°$. An RF phase comparator (Analog Devices AD8302, DC-2.7 GHz) is connected to Port 3 of Cells 3 and 4. The phase difference $\Delta\varphi_i$ of the two input ports is measured and a DC voltage $V_{\Delta\varphi}$ is sent out in real-time from the output port of the chip. The relationship between $V_{\Delta\varphi}$ and $\Delta\varphi_i$ is

$$\Delta\varphi_i\ [\text{deg}] = 180° - 100V_{\Delta\varphi}\ [\text{V}] \qquad \text{(S23)}$$

Combining Equations S18, S22 and S23, we can express the required B_{in} for unit cells in column m:

$$[B_{\text{in}}]_{10} = \text{round}\left(\frac{180-100V_{\Delta\varphi}}{5.625}m\right), \qquad \text{(S24)}$$

in which the operation "round" denotes rounding to the nearest integer. Finally, the required 6-digit input bit $[B_{\text{in}}]_2$ can also be obtained.

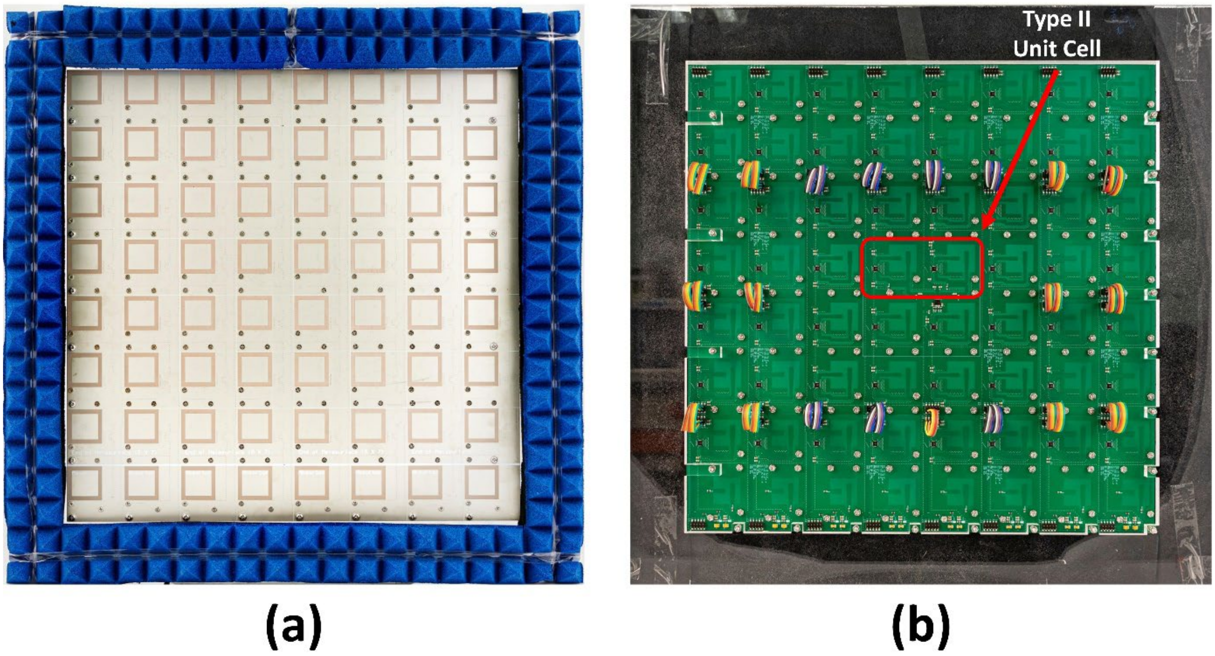

Figure S4. Fabricated normal reflection SRS. (a) Top and (b) bottom views of the fabricated SRS with 8 by 8 unit cells.

S5. Responding Time Analysis

Our SRS adapts to incident angle changes (up to 12 deg/s, as shown in Section 2.4.2) by detecting the phase difference $\Delta\varphi_i$ via a phase comparator (Analog Devices, AD8302), transmitting signals through microstrip lines, and adjusting phase shifters using an analog-to-digital converter (ADC) of the lookup table (LUT). To characterize the response time, in the following we analyze the delay contributions of each component:

- Phase comparator

According to the datasheet[3] of the phase comparator chip model AD8302, the chip responds in 40 ns for $\mathrm{d}\Delta\varphi_i = 15°$ change of the phase difference. Based on Equation S7 ($\theta_i = \sin^{-1}\left[\frac{\lambda}{2\pi D}\Delta\varphi_i\right]$), we can obtain:

$$\mathrm{d}\Delta\varphi_i = \frac{2\pi D}{\lambda}\cos\theta_i\,\mathrm{d}\theta_i \tag{S25}$$

With $D = 50$ mm, $\lambda = 125$ mm, and $\cos\theta_i < 1$ for θ_i within ± 50:

$$\mathrm{d}\Delta\varphi_i \leq \frac{2\pi \cdot 50}{125} \cdot 1 \cdot \mathrm{d}\theta_i \tag{S26}$$

In addition, the phase shifter (M/A-COM, MAPS-010164) has a 6-bit resolution, yielding a phase step of:

$$\mathrm{d}\Delta\varphi_i = 360°/2^6 = 5.625° \tag{S27}$$

Therefore, the minimum incident angle change to trigger a phase adjustment is:

$$\mathrm{d}\theta_i \geq \frac{125}{2\pi \cdot 50 \cdot 1} \times 5.625° \approx 2.24° \tag{S28}$$

Combining Equation S26 and S28, we can obtain $\mathrm{d}\Delta\varphi_i \leq 5.63°$. Therefore, the maximum responding time for the phase comparator chip is $t_1 = 5.63° \times 40\ \mathrm{ns}/15° = 15\ \mathrm{ns}$.

- Microstrip line

The total length of the microstrip line is around $l = 80\ \mathrm{mm}$, which will incur a propagation delay of $t_2 = l/v = 0.66\ \mathrm{ns}$, in which the constant $v = c/\sqrt{\epsilon_r}$ is the light speed in the substrate with $\epsilon_r = 6.15$.

- Analog-to-digital converter (ADC)

The ADC of LUT processing take $t_3 = 1\ \mu\mathrm{s}$, which dominates the time delay of the control circuit.

Therefore, the total system delay is approximately:

$$t_{\mathrm{total}} \approx 15\ \mathrm{ns} + 0.66\ \mathrm{ns} + 1\ \mu\mathrm{s} = 1.016\ \mu\mathrm{s} \tag{S29}$$

To ensure the phase shifter stability during the system delay, the theoretical upper limit of the angular velocity of the incident waves is $2.24°/1.016\ \mu\mathrm{s} = 2.2 \times 10^6$ deg/s. At 12 deg/s (1° in 83.3 ms), this ensures real-time performance, as the system adjusts thousands of times faster than the angle change rate, enabling stable operation with minimal latency. The control circuit, leveraging a simple MCU-based system (Section 4, Table 4), has the 1 μs ADC processing time step, which is the primary bottleneck but still negligible compared to the dynamics of 12 deg/s.

Practically, with a conservative total response of 1 ms (including unquantified delays like phase shifter switching) for 2.24° angular displacement, the system could handle ~2000 deg/s, far exceeding the tested 12 deg/s. Experimental results at 12 deg/s (Figure 5e) show stable reflection without significant power fluctuations, suggesting robustness beyond this rate. The upper dynamic limit depends on factors like phase shifter switching speed (not fully specified but typically ~10–100 ns for RF components like MAPS-010164), but our analysis indicates potential stability at thousands of degrees per second, pending further hardware characterization.

S6. Power Consumption Analysis

The SRS is designed for energy-efficient autonomous operation, leveraging a minimal hardware setup with 64 phase shifters (MAPS-010164), one phase comparator (AD8302), and a microcontroller unit (STM32F103VET6)-based lookup table (LUT). To address the power requirements of autonomous systems, we analyze the typical and maximum power consumption of each functional module, summarized in Table S4. These specifications are derived from the component datasheets[2-4] and validated with experimental measurements.

Table S4. Power Supply and Control Specifications of AD8302, MAPS-010164 and STM32F103VET6.

Module Series	Voltage Supply	Operation Current	Control Voltage	Control Current
MAPS-010164[2] (1 unit)	V_{CC}: +5.0 V<br>V_{EE}: -5.0 V	I_{CC}: 2.5 μA (Max.)<br>I_{EE}: 0.1 mA (Typ.)<br>1.0 mA (Max.)	V_H: 5.0 V<br>V_l: 0.0 V	1 μA<br>(Typ., per bit)
AD8302[3] (1 unit)	5.0 V	19 mA (Typ.)<br>25 mA (Max.)	N/A	N/A
STM32F103VET6[4]	3.3 V	40 mA (Min.)<br>50 mA (Max.)	N/A	N/A

S6.1 Power Consumption Calculation

- Lookup Table:

The lookup table (LUT) is stored in a microcontroller unit, with operation current of 40-50 mA under supplied voltage of 3.3 V and operation frequency 72 MHz. The maximum power consumption is:

$$P_1 = 3.3\ \mathrm{V} \times 50\ \mathrm{mA} = 165.0\ \mathrm{mW} \tag{S30}$$

- Phase shifters (64 units)

Typical: Each phase shifter consumes 0.52 mW (0.1 mA × 5 V + 2.5 μA × 5 V). For 64 units:

$$P_2 = 64 \times 0.52\ \mathrm{mW} \approx 33.3\ \mathrm{mW} \tag{S31}$$

Maximum: Each phase shifter consumes 5.02 mW (1.0 mA × 5 V + 2.5 μA × 5 V). For 64 units:

$$P_2 = 64 \times 5.02\ \mathrm{mW} \approx 321.3\ \mathrm{mW} \tag{S32}$$

- Control inputs (64 units × 6-bit):

Maximum: All 6 bits high:

$$P_3 = 64 \times 6 \times 5\ \mathrm{V} \times 1\ \mu\mathrm{A} = 1.92\ \mathrm{mW} \tag{S33}$$

- Phase compartor (1 unit):

Typical: 19 mA at 5 V:

$$P_4 = 5\ \mathrm{V} \times 19\ \mathrm{mA} \approx 95.0\ \mathrm{mW} \quad \text{(S34)}$$

Maximum: 25 mA at 5 V:

$$P_4 = 5\ \mathrm{V} \times 25\ \mathrm{mA} \approx 125.0\ \mathrm{mW} \quad \text{(S35)}$$

- Total power consumption:

Typical:

$$P_{\mathrm{total}} = 165.0 + 33.3 + 1.92 + 95.0 \approx 295.22\ \mathrm{mW} \quad \text{(S36)}$$

Maximum:

$$P_{\mathrm{total}} = 165.0 + 321.3 + 1.92 + 125.0 \approx 613.22\ \mathrm{mW} \quad \text{(S37)}$$

S6.2 Experimental Validation

In experiments, the SRS is powered by two DC voltage supplies (+5 V and -5 V), drawing measured currents of ~40 mA and ~10 mA, respectively. Including the dominated power consumed by the lookup table (P_1=165.0 mW), the actual power consumption is:

$$P_{\mathrm{meas.}} = 165\ \mathrm{mW} + 5\ \mathrm{V} \times (40\ \mathrm{mA} + 10\ \mathrm{mA}) = 415\ \mathrm{mW} \quad \text{(S38)}$$

which is higher than the calculated typical power (295.22 mW) but well below the maximum (613.22 mW), suggesting operation closer to typical conditions with additional losses (e.g., PCB inefficiencies, not included in calculations). The discrepancy may also arise from dynamic phase shifter operation or unaccounted control circuit.

S6.3 Discussion

The proposed SRS achieves an exceptionally low power consumption of 415 mW, with an average power per area of 2.6×10^{-4} W/cm^2, outperforming existing reconfigurable metasurfaces, such as Ma et al. (1.4×10^{-2} W/cm^2)[5] and Wang et al. (5.1×10^{-4} W/cm^2)[6], as shown in Table S5. This efficiency results from its simple structure (e.g. LUT, only 160 mW) and control logic, eliminating power-intensive components like FPGAs (around 5 W) or external sensors. The SRS's low power draw, combined with its autonomous LUT-based control, ensures energy efficiency and scalability for larger arrays, where doubling to 128 unit cells would further decrease the value of the average power per area.

Table S5. Power consumption comparison between the proposed SRS and existing works.

Work	Power Consumption (W)	Operation Frequency (GHz)	Number of Unit Cells	Total Area (cm^2)	Power per Area (W/cm^2)
Ma et al., 2019[4]	10.0	9.0	30×30	729	1.4×10^{-2}
Wang et al., 2024[5]	6.52	2.6	32×16	12800	5.1×10^{-4}
SRS	0.415	2.4	8×8	1600	2.6×10^{-4}

References

[1] F. Aieta, P. Genevet, M. A. Kats, N. Yu, R. Blanchard, Z. Gaburro, and F. Capasso, "Aberration-Free Ultrathin Flat Lenses and Axicons at Telecom Wavelengths Based on Plasmonic Metasurfaces," *Nano Letters,* vol. 12, no. 9, pp. 4932-4936, 2012.
[2] MACOM, "Digital Phase Shifter 6-Bit, 2.3 - 3.8 GHz,"
[3] Analog Devices, "RF/IF Gain and Phase Detector, LF - 2.7 GHz,"
[4] STMicroelectronics, "High-density performance line Arm®-based 32-bit MCU with 256 to 512KB Flash, USB, CAN, 11 timers, 3 ADCs, 13 communication interfaces,"
[5] Q. Ma, G. D. Bai, H. B. Jing, C. Yang, L. Li, and T. J. Cui, "Smart Metasurface with Self-Adaptively Reprogrammable Functions," Light Sci. Appl., vol. 8, no. 1, p. 98, 2019.
[6] J. Wang, W. Tang, J. C. Liang, L. Zhang, J. Y. Dai, X. Li, S. Jin, Q. Cheng and T. J. Cui, "Reconfigurable intelligent surface: Power consumption modeling and practical measurement validation," IEEE Transactions on Communications, 2024.
